# Mind the gap: Exploring differences in suicide literacy between cybersuicide and offline suicide

**DOI:** 10.3389/fpubh.2022.1061590

**Published:** 2023-01-16

**Authors:** Ang Li, Dongdong Jiao

**Affiliations:** ^1^Department of Psychology, Beijing Forestry University, Beijing, China; ^2^National Computer System Engineering Research Institute of China, Beijing, China

**Keywords:** suicide literacy, cybersuicide, psycholinguistic analysis, LIWC, social media

## Abstract

**Introduction:**

The highly public nature of cybersuicide contradicts long-held beliefs of offline suicide, which may cause differences in the way people perceive and respond to both of them. However, knowledge of whether and how suicide literacy differs between cybersuicide and offline suicide is limited.

**Methods:**

By analyzing social media data, this paper focused on livestreamed suicide and aimed to compare suicide literacy between cybersuicide and offline suicide on three aspects, including false knowledge structure, extent of association with stigma, and linguistic expression pattern. 7,236 Sina Weibo posts with relevant keywords were downloaded and analyzed. First, a content analysis was performed by human coders to determine whether each post reflected suicide-related false knowledge and stigma. Second, a text analysis was conducted using the Simplified Chinese version of LIWC software to automatically extract psycholinguistic features from each post. Third, based on selected features, classification models were developed using machine learning techniques to differentiate false knowledge of cybersuicide from that of offline suicide.

**Results:**

Results showed that, first, cybersuicide-related posts generally reflected more false knowledge than offline suicide-related posts ($\chi_{1}^{2}=$255.13, *p* < 0.001). Significant differences were also observed in seven false knowledge types. Second, among posts reflecting false knowledge, cybersuicide-related posts generally carried more stigma than offline suicide-related posts ($\chi_{1}^{2}$ = 116.77, *p* < 0.001). Significant differences were also observed in three false knowledge types. Third, among established classification models, the highest F1 value reached 0.70.

**Discussion:**

The findings provide evidence of differences in suicide literacy between cybersuicide and offline suicide, and indicate the need for public awareness campaigns that specifically target cybersuicide.

## 1. Introduction

Mental health literacy refers to “knowledge and beliefs about mental disorders which aid their recognition, management or prevention” (1). Low mental health literacy is known to negatively affect health outcomes for people with mental disorders, particularly when stigma is also present (2, 3). Promising practices for improving mental health literacy should be designed to suit the targeted audience (4). Therefore, it is important to understand the content and nature of false knowledge and beliefs that people hold about specific mental disorders.

Suicide is one of the leading causes of death across all ages, especially among adolescents (5, 6). Suicide itself is not a specific mental disorder, but one of the most important factors that cause suicide is mental disorders. Improving suicide literacy (public knowledge about the causes, risk factors, treatment and prevention of suicide) may lead to better outcomes for those at suicide risk. In recent decades, the development of internet creates a new form of suicide that covers a variety of internet-mediated suicidal behaviors and phenomena (i.e., cybersuicide) (7). Unlike the traditional form of suicide that is not influenced by the internet (i.e., offline suicide), cybersuicide enables suicidal people to share suicidal thoughts and behaviors with their online social networks, and allows real time interaction between suicidal people and their audience. Therefore, the highly public nature of cybersuicide contradicts long-held beliefs of offline suicide that suicide should be a personal and private action (8, 9). This contradiction implies the internet is changing the context and socio-cultural norms of death-related behaviors and phenomena, which may influence the way people perceive and respond to suicide (10, 11).

In recent years, considerable research effort has been devoted to investigating public reaction to cybersuicide. Results showed that cybersuicide carries more stigma than offline suicide (12–15). Besides, significant differences were also found between cybersuicide and offline suicide in linguistic pattern of stigmatizing expressions (12). These findings provide evidence that the public reacts differently to cybersuicide and offline suicide. However, knowledge of whether and how suicide literacy differs between cybersuicide and offline suicide is still very limited.

To address this concern, by analyzing Chinese social media data, this study purposed to systematically investigate differences in suicide literacy between cybersuicide and offline suicide on three aspects, including false knowledge structure, extent of association with stigma, and linguistic expression pattern.

It is worth noting here that, in China, livestreamed suicide is one of the most common forms of cybersuicide (16, 17). Therefore, it is expected that the public should feel more familiar with livestreamed suicide than other forms of cybersuicide, and subsequently should be more likely to express their opinions about livestreamed suicide on social media. Therefore, in order to collect sufficient cybersuicide-related social media data, this study mainly focused on livestreamed suicide and compared this representative form of cybersuicide with offline suicide.

## 2. Materials and methods

**Figure 1:**
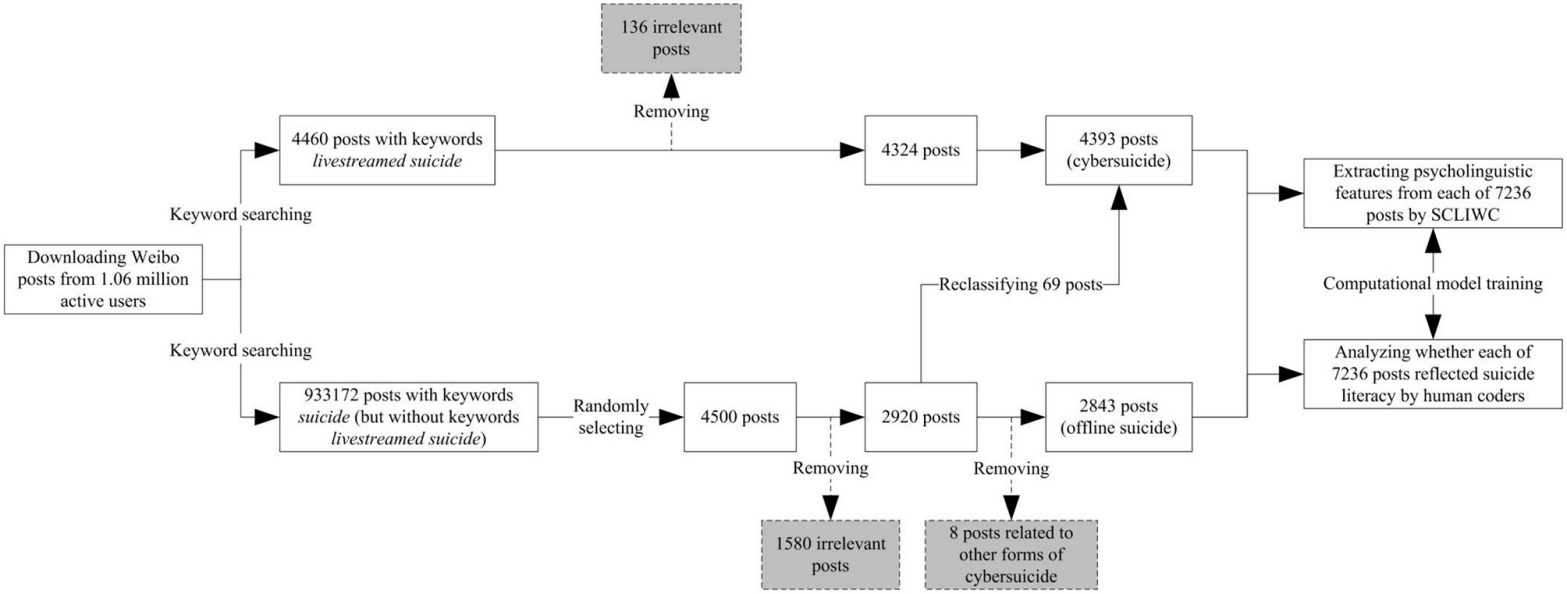
The research process.

Research process of this study consisted of three stages, including (i) data collection, (ii) data preprocessing, and (iii) data analysis. All data were obtained from Sina Weibo, a popular Chinese microblogging website. Research process is shown in Figure 1.

This study was reviewed and approved by the Institutional Review Board of the Institute of Psychology, Chinese Academy of Sciences (protocol number: H15009). Because this study collected and analyzed publicly available social media data and involved no personally identifiable data, the ethics committee waived the requirement of written informed consent for participation.

### 2.1. Data collection

Weibo posts with relevant keywords were obtained from a self-established dataset, which was composed of 1.06 million active Weibo users and all their publicly available Weibo posts since registration (18). Keywords indicating cybersuicide and offline suicide were selected respectively, including “livestreamed suicide” (直播自杀, 自杀直播) and “suicide” (自杀). It is worth noting here that, in China, offline suicide is much more prevalent than cybersuicide. Therefore, unless otherwise specified, this term “suicide” is commonly used to refer to offline suicide in China.

In May 2020, selected keywords were used to search the dataset and download 4,460 posts with keywords “livestreamed suicide” and 933,172 posts with keywords “suicide” (but without keywords “livestreamed suicide”), respectively.

### 2.2. Data preprocessing

After data collection, data preprocessing was performed to transform raw data into quality data that is ready for further analysis.

First, 4,500 posts were randomly selected from offline suicide group to balance the number of posts between cybersuicide group and offline suicide group (cybersuicide: 4,460 posts; offline suicide: 4,500 posts).

Second, on downloaded posts, manual scrutiny of irrelevant posts was performed by an experienced researcher. In this study, irrelevant posts were defined as those describing suicide in fiction works and animals (e.g., movies and pets) or talking about suicide without suicidal purposes (e.g., making jokes). After removal of 1,716 irrelevant posts, there remained 7,244 posts (cybersuicide: 4,324 posts; offline suicide: 2,920 posts).

Third, during manual scrutiny of irrelevant posts, 77 mislabeled posts from offline suicide group were reclassified as cybersuicide-related posts. Among these mislabeled posts, 69, 4, 2, and 2 posts were related to livestreamed suicide, suicide “game”, prosuicide website and forum, and internet suicide pact, respectively. Because this study exclusively focused on livestreamed suicide, eight posts associated with other three forms of cybersuicide were not involved in further data analysis. Finally, in this study, the sample size reached 7,236 posts, including 4,393 cybersuicide-related posts (men: 2,473 posts; women: 1,920 posts) and 2,843 offline suicide-related posts (men: 1,589 posts; women: 1,254 posts).

Fourth, text analysis was conducted using the Simplified Chinese version of LIWC software (SCLIWC) to automatically extract psycholinguistic features from each of 7,236 posts. SCLIWC is a lexicon-based tool that aggregates individual words into semantic and syntactic categories (19). For each post, after computation of word frequency in psychologically meaningful categories, standardized values of psycholinguistic features were estimated accordingly.

### 2.3. Data analysis

In this study, data analyses were carried out in two phases, including human coding and computational model training. Specifically, in the phase of human coding, analyses were made for exploring differences in false knowledge structure and extent of association with stigma between cybersuicide and offline suicide; while in the phase of computational model training, analyses were made for exploring differences in linguistic expression pattern between cybersuicide and offline suicide.

#### 2.3.1. Human coding

All 7,236 posts were analyzed manually by two independent human coders to determine whether each post reflected suicide-related false knowledge and stigma. The coding framework of suicide literacy was based on available evidence and expert consensus, which finally contained 11 items from Calear's Literacy of Suicide Scale (LOSS) (20) (Table 1). Details on coding framework and coding results of stigma can be found in another paper (12).

**Table 1:** Results of human coding for suicide literacy: *n* = 863.

**False knowledge types**	**Unbalanced dataset**		**Balanced dataset**	
	**Cybersuicide**	**Offline suicide**	**Cybersuicide**	**Offline suicide**
	**(*****n***=**739)**	**(*****n***=**124)**	**(*****n***=**76)**	**(*****n***=**76)**
1 “Unpreventable”: nothing can be done to stop people from making the attempt once they have made up their minds to kill themselves	9 (1.22%)	2 (1.61%)	2 (2.63%)	2 (2.63%)
2 “Should keep secrets”: people who have thoughts about suicide should not tell others about it	176 (23.82%)	16 (12.90%)	16 (21.05%)	16 (21.05%)
3 “Well-planned”: all people who attempt suicide plan their attempt in advance	4 (0.54%)	49 (39.52%)	4 (5.26%)	4 (5.26%)
4 “No future plans”: most people who suicide don't make future plans	10 (1.35%)	7 (5.65%)	7 (9.21%)	7 (9.21%)
5 “Must ‘succeed”': very few people who attempt suicide fail to kill themselves	51 (6.90%)	4 (3.23%)	4 (5.26%)	4 (5.26%)
6 “Manipulating/attracting attention”: those who attempt suicide do so only to manipulate others and attract attention to themselves	380 (51.42%)	23 (18.55%)	23 (30.26%)	23 (30.26%)
7 “Mentally ill”: a person who suicides is mentally ill	3 (0.41%)	6 (4.84%)	3 (3.95%)	3 (3.95%)
8 “Depressed”: if assessed by a psychiatrist, everyone who suicides would be diagnosed as depressed	7 (0.95%)	6 (4.84%)	6 (7.89%)	6 (7.89%)
9 “No change of mind”: people who want to attempt suicide cannot change their mind quickly	26 (3.52%)	7 (5.65%)	7 (9.21%)	7 (9.21%)
10 “Stop media coverage of suicide”: media coverage of suicide will inevitably encourage other people to attempt suicide	72 (9.74%)	3 (2.42%)	3 (3.95%)	3 (3.95%)
11 “Waiting for experts”: only experts can help people who want to suicide	1 (0.14%)	1 (0.81%)	1 (1.32%)	1 (1.32%)

The IBM SPSS Statistics software (SPSS, version 20) was used to analyze statistics below. The agreement between pairs of human coders was measured by Cohen *k* coefficient, and any disagreement was resolved by a researcher. Besides, between cybersuicide and offline suicide, the differences in proportions of posts reflecting suicide literacy were estimated by Pearson's chi-square test and Fisher's exact test.

#### 2.3.2. Computational model training

A series of machine learning models were trained to differentiate false knowledge of cybersuicide from that of offline suicide. Within the training process, human coding results and psycholinguistic feature values were considered as the ground truth and the predictors, respectively.

For the ground truth, in this study, class imbalance problem existed. Specifically, between cybersuicide and offline suicide, obvious differences were observed in amounts of both general false knowledge and specific false knowledge types. In machine learning, when the dataset is extremely imbalanced, most existing classification algorithms may not perform well on minority class. To resolve this concern and obtain a well-balanced dataset, at the level of specific false knowledge types, data from the majority class were randomly eliminated until there were as many data in both classes. The balanced dataset composed of 152 posts is shown in Table 1.

For the predictors, to improve the predictive performance of models, psycholinguistic features that were valid for differentiating suicide literacy between cybersuicide and offline suicide were selected as key features. It is worth noting here that, to avoid overfitting in computational model training, the data used for feature selection remained independent of the data used for model training. Specifically, based on a random selection of 15 posts from each class of the balanced dataset, a series of two-tailed independent *t* tests were performed by the SPSS software to compare values of psycholinguistic features between cybersuicide and offline suicide, and then effect sizes (Cohen *d* coefficient) were computed from the estimated *t* values by the Effect Size Calculators (https://lbecker.uccs.edu/). Features that were statistically significant at 0.05 and had a Cohen *d* > 0.20 or <-0.20 were considered as key features.

Finally, based on the remaining 122 posts from the balanced dataset (152–30 = 122), a series of classification models were built on selected key features. The Waikato Environment for Knowledge Analysis software (WEKA, version 3.9.4) was used to train machine learning models. By using the five-fold cross-validation technique, the classification performance of models was evaluated in terms of precision, recall, and F1.

## 3. Results

### 3.1. Human coding

The Cohen *k* coefficients for “general false knowledge” and “specific false knowledge types” reached 0.91 and 0.87, respectively, reflecting almost perfect agreement. Results of human coding for suicide literacy are shown in Table 1.

**Figure 2:**
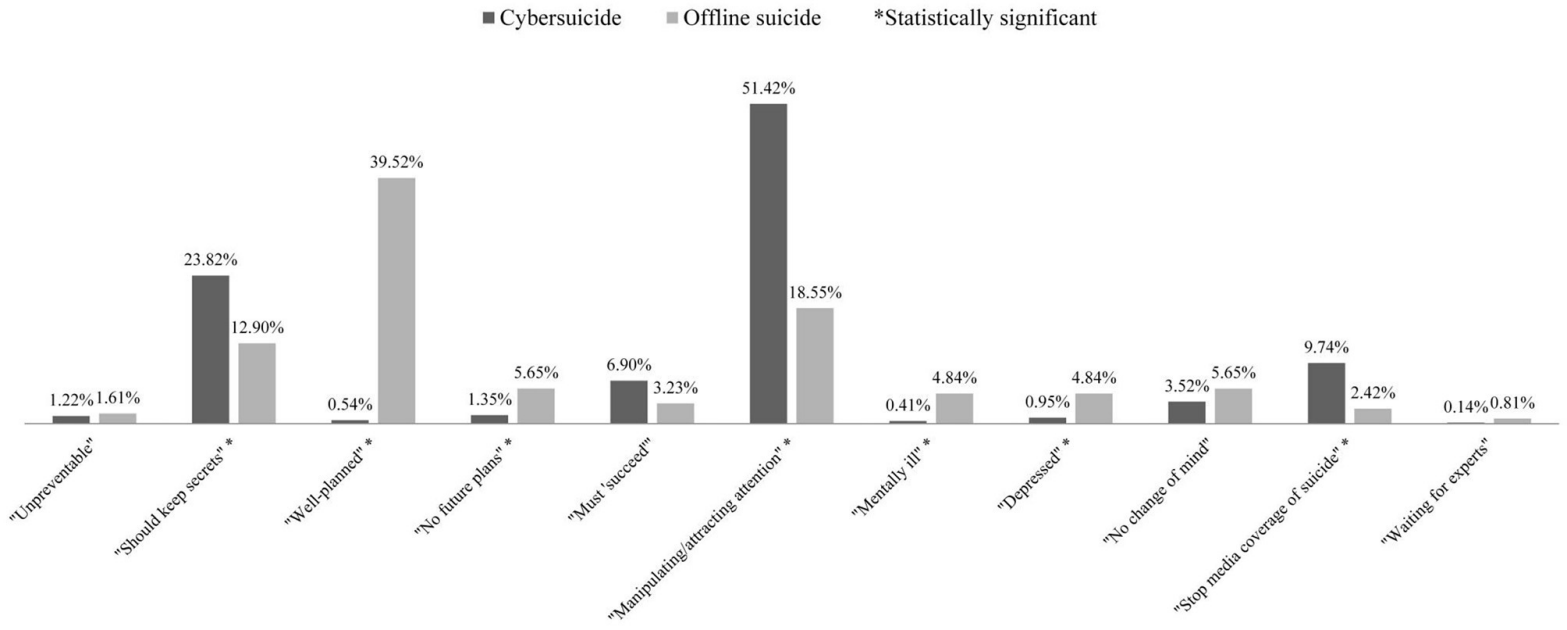
Comparison of proportions of posts indicating specific false knowledge types between cybersuicide and offline suicide. *Statistically significant.

First, for differences in false knowledge structure, cybersuicide-related posts generally reflected more false knowledge than offline suicide-related posts (cybersuicide: 739/4,393, 16.82%; offline suicide: 124/2,843, 4.36%; $\chi_{1}^{2}=$255.13, *p* < 0.001). Furthermore, between cybersuicide and offline suicide, significant differences were also observed in specific false knowledge types (Fisher exact test *p* < 0.001) (Figure 2). Specifically, cybersuicide-related posts were more likely than offline suicide-related posts to be coded as three false knowledge types, including “Should keep secrets” (cybersuicide: 176/739, 23.82%; offline suicide: 16/124, 12.90%; $\chi_{1}^{2}=$7.31, *p* < 0.01), “Manipulating/attracting attention” (cybersuicide: 380/739, 51.42%; offline suicide: 23/124, 18.55%; $\chi_{1}^{2}=$46.10, *p* < 0.001), and “Stop media coverage of suicide” (cybersuicide: 72/739, 9.74%; offline suicide: 3/124, 2.42%; $\chi_{1}^{2}=$7.18, *p* < 0.01); while offline suicide-related posts were more likely than cybersuicide-related posts to be coded as four false knowledge types, including “Well-planned” (cybersuicide: 4/739, 0.54%; offline suicide: 49/124, 39.52%; $\chi_{1}^{2}=$279.82, *p* < 0.001), “No future plans” (cybersuicide: 10/739, 1.35%; offline suicide: 7/124, 5.65%; Fisher exact test *p* < 0.01), “Mentally ill” (cybersuicide: 3/739, 0.41%; offline suicide: 6/124, 4.84%; Fisher exact test *p* < 0.001), and “Depressed” (cybersuicide: 7/739, 0.95%; offline suicide: 6/124, 4.84%; Fisher exact test *p* < 0.01). Besides, cybersuicide-related posts were often coded as “Manipulating/attracting attention” (51.42%) and “Should keep secrets” (23.82%); while offline suicide-related posts were often coded as “Well-planned” (39.52%).

Similar results were also found for different genders. Cybersuicide-related posts generally reflected more false knowledge than offline suicide-related posts for both of men (cybersuicide: 369/2,473, 14.92%; offline suicide: 66/1,589, 4.15%; $\chi_{1}^{2}$ = 117.30, *p* < 0.001) and women (cybersuicide: 370/1,920, 19.27%; offline suicide: 58/1,254, 4.63%; $\chi_{1}^{2}$ = 139.47, *p* < 0.001). Furthermore, between cybersuicide and offline suicide, significant differences in specific false knowledge types were also observed for both of men (Fisher exact test *p* < 0.001) and women (Fisher exact test *p* < 0.001). Specifically, for men, cybersuicide-related posts were more likely than offline suicide-related posts to be coded as three false knowledge types, including “Should keep secrets” (cybersuicide: 89/369, 24.12%; offline suicide: 4/66, 6.06%; $\chi_{1}^{2}$ = 10.86, *p* < 0.01), “Manipulating/attracting attention” (cybersuicide: 194/369, 52.57%; offline suicide: 11/66, 16.67%; $\chi_{1}^{2}$ = 28.97, *p* < 0.001), and “Stop media coverage of suicide” (cybersuicide: 42/369, 11.38%; offline suicide: 1/66, 1.52%; $\chi_{1}^{2}$ = 6.12, *p* < 0.05); while offline suicide-related posts were more likely than cybersuicide-related posts to be coded as four false knowledge types, including “Well-planned” (cybersuicide: 1/369, 0.27%; offline suicide: 35/66, 53.03%; $\chi_{1}^{2}=$205.30, *p* < 0.001), “No future plans” (cybersuicide: 3/369, 0.81%; offline suicide: 3/66, 4.55%; Fisher exact test *p* < 0.05), “Mentally ill” (cybersuicide: 0/369, 0%; offline suicide: 2/66, 3.03%; Fisher exact test *p* < 0.05), and “Depressed” (cybersuicide: 4/369, 1.08%; offline suicide: 4/66, 6.06%; Fisher exact test *p* < 0.05). For women, cybersuicide-related posts were more likely than offline suicide-related posts to be coded as “Manipulating/attracting attention” (cybersuicide: 186/370, 50.27%; offline suicide: 12/58, 20.69%; $\chi_{1}^{2}=$17.65, *p* < 0.001); while offline suicide-related posts were more likely than cybersuicide-related posts to be coded as three false knowledge types, including “Well-planned” (cybersuicide: 3/370, 0.81%; offline suicide: 14/58, 24.14%; Fisher exact test *p* < 0.001), “No future plans” (cybersuicide: 7/370, 1.89%; offline suicide: 4/58, 6.90%; Fisher exact test *p* < 0.05), and “Mentally ill” (cybersuicide: 3/370, 0.81%; offline suicide: 4/58, 6.90%; Fisher exact test *p* < 0.01).

**Figure 3:**
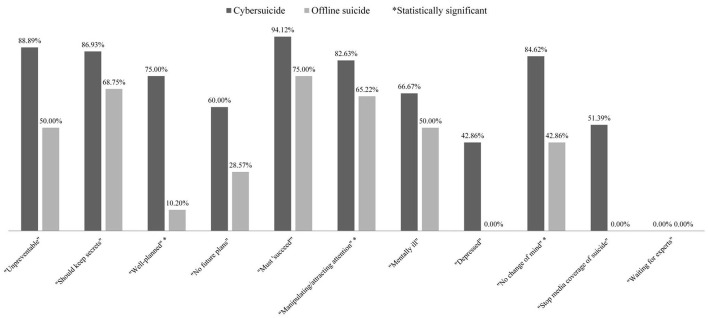
Comparison of proportions of stigmatizing posts between cybersuicide and offline suicide across specific false knowledge types. *Statistically significant.

Second, for differences in extent of association with stigma, among posts reflecting false knowledge, cybersuicide-related posts generally reflected more stigma than offline suicide-related posts (cybersuicide: 596/739, 80.65%; offline suicide: 43/124, 34.68%; $\chi_{1}^{2}$ = 116.77, *p* < 0.001) (Table 2). Furthermore, cybersuicide-related posts were more likely than offline suicide-related posts to be coded as stigmatizing across three specific false knowledge types (Figure 3), including “Well-planned” (cybersuicide: 3/4, 75.00%; offline suicide: 5/49, 10.20%; Fisher exact test *p* < 0.01), “Manipulating/attracting attention” (cybersuicide: 314/380, 82.63%; offline suicide: 15/23, 65.22%; Fisher exact test *p*=0.05), and “No change of mind” (cybersuicide: 22/26, 84.62%; offline suicide: 3/7, 42.86%; Fisher exact test *p* < 0.05).

**Table 2:** Proportions of stigmatizing posts across different false knowledge types: *n* = 863.

**False knowledge types**	**Cybersuicide**		**Offline suicide**	
	**(*****n*** = **739)**		**(*****n*** = **124)**	
	**Stigma**	**Non-stigma**	**Stigma**	**Non-stigma**
1 “Unpreventable”	8 (88.89%)	1 (11.11%)	1 (50.00%)	1 (50.00%)
2 “Should keep secrets”	153 (86.93%)	23 (13.07%)	11 (68.75%)	5 (31.25%)
3 “Well-planned”	3 (75.00%)	1 (25.00%)	5 (10.20%)	44 (89.80%)
4 “No future plans”	6 (60.00%)	4 (40.00%)	2 (28.57%)	5 (71.43%)
5 “Must ‘succeed”'	48 (94.12%)	3 (5.88%)	3 (75.00%)	1 (25.00%)
6 “Manipulating/attracting attention”	314 (82.63%)	66 (17.37%)	15 (65.22%)	8 (34.78%)
7 “Mentally ill”	2 (66.67%)	1 (33.33%)	3 (50.00%)	3 (50.00%)
8 “Depressed”	3 (42.86%)	4 (57.14%)	0 (0%)	6 (100%)
9 “No change of mind”	22 (84.62%)	4 (15.38%)	3 (42.86%)	4 (57.14%)
10 “Stop media coverage of suicide”	37 (51.39%)	35 (48.61%)	0 (0%)	3 (100%)
11 “Waiting for experts”	0 (0%)	1 (100%)	0 (0%)	1 (100%)

Similar results were also found for different genders. Among posts reflecting false knowledge, cybersuicide-related posts generally reflected more stigma than offline suicide-related posts for both of men (cybersuicide: 291/369, 78.86%; offline suicide: 13/66, 19.70%; $\chi_{1}^{2}=$93.12, *p* < 0.001) and women (cybersuicide: 305/370, 82.43%; offline suicide: 30/58, 51.72%; $\chi_{1}^{2}$ = 27.80, *p* < 0.001). Furthermore, for men, cybersuicide-related posts were more likely than offline suicide-related posts to be coded as stigmatizing across two specific false knowledge types, including “Must ‘succeed”' (cybersuicide: 24/24, 100%; offline suicide: 0/1, 0%; Fisher exact test *p* < 0.05) and “Manipulating/attracting attention” (cybersuicide: 159/194, 81.96%; offline suicide: 5/11, 45.45%; Fisher exact test *p* < 0.05).

### 3.2. Computational model training

Based on randomly selected 30 posts from the balanced dataset, five key features were selected for model training (Table 3). Based on the remaining 122 posts from the balanced dataset, by using five different machine learning algorithms (Logistic Regression, Support Vector Machine, Multilayer Perceptron Neutral Network, C4.5 Decision Tree, and SPAARC Decision Tree), a series of classification models were built on key features. Results showed that the SPAARC Decision Tree model had the best classification performance (precision: 0.82; recall: 0.72; F1: 0.70) (Table 4).

**Table 3:** Key features selection: *n* = 30.

**Key features**	***t* (*df*)**	***p***	**Cohen *d***
Signal words for past tense	2.39 (28)	< 0.05	0.90
Signal words for present tense	2.72 (28)	< 0.05	1.03
Family	−2.14 (28)	< 0.05	−0.81
Time	3.05 (28)	< 0.01	1.15
Leisure	4.68 (28)	< 0.001	1.77

**Table 4:** Performance of classification models: *n* = 122.

**Classification models**	**Precision**	**Recall**	**F1**
Logistic Regression	0.63	0.63	0.63
Support Vector Machine	0.62	0.62	0.62
Multilayer Perceptron Neutral Network	0.68	0.68	0.68
C4.5 Decision Tree	0.80	0.71	0.69
SPAARC Decision Tree	0.82	0.72	0.70

## 4. Discussion

### 4.1. Principal findings

According to our knowledge, this is the first study that compares suicide literacy between cybersuicide and offline suicide directly and systematically. Findings of this study provide evidence for understanding the content and nature of false knowledge and beliefs that people hold about different forms of suicide, and also provide insights for designing future public awareness campaigns.

First, in terms of false knowledge structure, the public has different interpretations and reactions to cybersuicide and offline suicide. Results showed that cybersuicide (16.82%) carried more false knowledge and beliefs than offline suicide (4.36%), reflecting a dire need for improving suicide literacy about cybersuicide. Furthermore, between cybersuicide and offline suicide, significant differences were also observed in proportions of specific false knowledge types, reflecting the differences in weights assigned to the prevalence of false knowledge types. For example, cybersuicide was more likely than offline suicide to be associated with three false knowledge types, including “Should keep secrets”, “Manipulating/attracting attention”, and “Stop media coverage of suicide”, which may be due to the prevalence of a distinctive stigmatizing stereotype (false representation stigma, a belief that people livestreaming their suicides do not really want to kill themselves) influenced by the highly public and interactive nature of cybersuicide (12). Similar results were also found for different genders. It implies that, as a social action, cybersuicide may have a different structure from offline suicide as a private action (8), which could facilitate the creation of emerging socio-cultural contexts and norms surrounding cybersuicide. Therefore, the general public is in dire need of guidelines on how people can perceive, communicate, and respond to cybersuicide appropriately.

Second, in terms of extent of association with stigma, cybersuicide and offline suicide have different priorities for raising the public awareness. Results showed that, among posts with false knowledge, cybersuicide-related posts (80.65%) generally carried more stigma than offline suicide-related posts (34.68%), reflecting the public reacts strongly to cybersuicide (13–15). Furthermore, between cybersuicide and offline suicide, significant differences were also observed in proportions of stigma across different false knowledge types, reflecting the differences in priorities for improvement. Similar results were also found for different genders, except that an additional significant difference was found in “Must succeed” only for posts by men. In specific, for men, cybersuicide-related posts were more likely than offline suicide-related posts to be coded as stigmatizing in the false knowledge type “Must succeed”. This inconsistency may be associated with greater glorification of cybersuicide by men (21). Because obvious differences existed in prevalence and dangerousness of false knowledge types between cybersuicide and offline suicide, public awareness campaigns should be designed to target specific form of suicide rather than to combine and confront different forms of suicide as a whole. For cybersuicide, in the future, literacy promotion efforts should be made to correct those more widespread and dangerous false knowledge types, like “Should keep secrets” and “Manipulating/attracting attention”.

Third, in terms of linguistic expression pattern, cybersuicide and offline suicide have different ways of expressing false knowledge. Results showed that, between cybersuicide and offline suicide, significant differences existed in psycholinguistic features of suicide literacy expressions. For example, suicide literacy expressions of cybersuicide were associated with more frequent use of words related to leisure (e.g., chat) and time (e.g., end), which may be attributed to the reality of real-time and long-lasting interaction between suicidal people and their audience. By contrast, suicide literacy expressions of offline suicide were associated with more frequent use of family-related words (e.g., baby, parent, husband), which may be attributed to public comments about the harmful effect of suicide death on family, friends and others in suicidal people's social networks. The reason for less frequent use of family-related words in cybersuicide-related posts may be due to the prevalent misbelief that cybersuicide is not real (21). Recent studies suggested the highly prevalence and spread of health misinformation on the internet (22, 23), and confirmed the potential for using new media to achieve improved health outcomes (24–26). Therefore, sufficient understanding in different ways of expressing false knowledge could make the automatic detection of suicide literacy expressions more targeted and improve the delivery of tailored messages for health promotion.

### 4.2. Limitations

Findings of this study may have limited generalizability. First, this study exclusively focused on livestreamed suicide and did not involve other forms of cybersuicide. Therefore, it remains unknown whether findings of this study can be applicable to other forms of cybersuicide. Second, social media users are not representative of all people in the real world. Findings of this study need to be further examined with more diverse populations in the future.

## 5. Conclusion

By analyzing social media data, this study compared suicide literacy between cybersuicide and offline suicide directly and systematically. The findings provide evidence of differences in suicide literacy between cybersuicide and offline suicide, and indicate the need for designing public awareness campaigns that specifically target cybersuicide.

## Data availability statement

The raw data supporting the conclusions of this article will be made available by the authors, without undue reservation.

## Ethics statement

The studies involving human participants were reviewed and approved by Institutional Review Board of the Institute of Psychology, Chinese Academy of Sciences. Written informed consent for participation was not required for this study in accordance with the national legislation and the institutional requirements.

## Author contributions

AL contributed to conception and design of the study, interpreted psychological results, and wrote the first draft of the manuscript. AL and DJ collected data and performed the statistical analyses. Both authors took responsibility for the integrity of the data, the accuracy of the data analysis, contributed to the article, and approved the submitted version.
